# The science behind the sweetness in our diets

**DOI:** 10.2471/BLT.14.031114

**Published:** 2014-11-01

## Abstract

Jim Mann tells Fiona Fleck what happened when WHO applied a rigorous new method of scientific evaluation to its guideline recommending that we keep our intake of “free sugars” in food and drink to less than 10% of dietary intake.

Q: What are “free sugars”?

A: According to WHO, the term “free sugars” refers to all monosaccharides and disaccharides added to foods by the manufacturer, cook or consumer, plus the sugars that are naturally present in honey, syrups and fruit juices. Monosaccharides have one sugar molecule and include glucose, galactose and fructose. Disaccharides have two molecules. The most widely consumed disaccharide is sucrose or table sugar.

Q: So the recommendations don’t apply to other types of sugar?

A: That’s right. The WHO recommendations only apply to free sugars. These do not include the sugars present in whole fruit and vegetables, which are sometimes known as intrinsic sugars. These sugars are encapsulated by a plant cell wall. They tend to be digested more slowly and take longer to enter the blood stream than free sugars.

“The WHO recommendations only apply to free sugars. These do not include the sugars present in whole fruit and vegetables.”

Q: Food labels don’t refer to “free sugars”, but “added sugar”, what is that?

A: The term “added sugar” is widely used in the United States and some other countries – although there is no universally agreed definition of “added sugar”. For the most part the term “added sugar” describes the same group of sugars as free sugars, but the term “free sugars” is more precise. For example, it is unclear whether concentrated fruit juice contains added sugar while there is no doubt that it contains free sugars. WHO decided that a more precise definition was needed for the purpose of guidelines and developed the definition of free sugars. The term “free sugars” is becoming more widely used. The recent draft report from the Specialist Advisory Committee on Nutrition to the United Kingdom government has also recommended use of the term. There are other unhelpful terms when it comes to describing sugars, for example: raw sugar, unrefined sugar and natural sugar. These are all free sugars.

Q: Why was the guideline updated?

A: The Organization made a clear recommendation in number 916 of its Technical Report series in 2003 that our consumption of free sugars should account for less than 10% of our dietary intake. The food industry and some countries, particularly the United States, questioned very strongly whether WHO had good enough evidence for this and doubts have lingered since then. When WHO decided to update the guideline, as part of WHO’s nutrition and dietary guidelines, the Nutrition Guidance Expert Advisory Group (the body of experts responsible for advising WHO on nutrition guidelines) was asked to answer two questions: what are the health effects of the consumption of free sugars and has any evidence emerged since 2003 to suggest that the existing recommendation (to keep the intake of free sugars below 10% of total energy) should be revised? Our guidance group decided to request two systematic reviews to help answer these questions focusing on the health outcomes, which they identified as the two priorities for this work: one on dental caries (tooth decay) and one on unhealthy weight gain (i.e. overweight and obesity).

Q: Why just those two?

A: There are more data on the effect of free sugars on dental caries than for other NCDs. Dental caries are not only a very unpleasant condition for the person affected, but treatment of caries consumes 6 to 10% of health-care budgets worldwide. Obesity was our other focus because everyone now acknowledges that we have a global epidemic of obesity and that obesity drives other NCDs – type 2 diabetes, certain cancers (e.g. post-menopausal breast cancer, colorectal cancer) and, to some extent, cardiovascular disease. It would have been interesting to examine the effect of sugars on cardiovascular disease, other NCDs and their risk factors, but the Nutrition Guidance Expert Advisory Group believed that focusing on dental caries and obesity would be sufficient.

Q: How did you set about conducting the systematic review on free sugars and obesity?

A: We focused on answering four questions: does decreasing the intake of free sugars reduce body weight and does increasing free sugars result in increased body weight? Each of these questions was examined separately in adults and in children. We set criteria for the studies that we would include in our search for the answers, such as: was the study of an appropriate design? Was dietary intake measured appropriately? Were the studies done in an unbiased way? Two types of studies were included: first, randomized controlled trials that involved asking participants to alter their usual sugar intake, so that the effects of increasing or decreasing their intake could be compared with a control group, who maintained their usual intake; and second, cohort studies, which involved following people with known intakes of free sugars or sugar-sweetened drinks to determine the extent to which consumption influences long-term weight outcomes. We started by searching databases with keywords and combing through the scientific literature for every conceivably relevant research publication. Then we had to put the different sets of results together so that a much clearer picture emerged than could be obtained by looking at the studies individually. Three researchers worked for almost a year on this and, of course, others were involved too. We started with 17 000 research papers, but, after applying the criteria, we narrowed down our selection to 68. Then we did a meta-analysis of the 68 to produce the strongest and most up-to-date evidence.

Q: In what way were your team’s findings different to those a decade earlier, which formed the evidence base for the 2003 recommendations on free sugars intake? Were these changes due to applying the grading of recommendations assessment, development and evaluation (GRADE) method, due to differences in the newly emerged evidence, or both?

A: When using the GRADE method, which WHO now uses for evaluating the strength of evidence which will serve as the basis for making recommendations, we found very convincing high-grade evidence, based on the effect of free sugars on body weight and dental caries, to show that the intake of free sugars should be kept below 10% of total energy intake. So our findings re-affirm the 2003 recommendation – that individuals should keep their free sugars intake to less than 10% – and provide evidence that a further reduction of free sugars to 5% of total energy intake may confer additional health benefits.

Q: As you said, there were lingering doubts about the strength of the evidence for the 2003 guideline, propagated by the food industry and some countries. But now, ironically, the evidence that has emerged since 2003 has not only reinforced the earlier 10% recommendation, but provided the basis for a much tougher recommendation of keeping free sugars at less than 5% of total energy intake. What are the implications of this affirmation of the earlier recommendation?

A: It is immensely reassuring for all health professionals and, indeed, for the general public to hear the strong reinforcement and potential strengthening of this message. Further reinforcement recently came from another highly regarded body, the Specialist Advisory Committee on Nutrition in the United Kingdom in a draft report on carbohydrates released in July, which includes remarkably similar advice on free sugars to that of WHO and, further reinforcing the message, the two sets of recommendations were developed independently of each other.

Q: If countries follow the draft WHO recommendations, what contribution could this make to “halting the rise of diabetes and obesity”, one of the goals in the Global Action Plan for the Prevention and Control of Noncommunicable Diseases?

A: We don’t really know because no country has made a serious attempt to implement such recommendations. An enormous body of evidence, however, suggests that reduction in the intake of energy dense foods (which are often high in fat and free sugars), and of sugar sweetened beverages is almost certain to halt the epidemic of obesity and to reduce the risk of type 2 diabetes and other related NCDs. The question is how best to implement the recommendations. A whole raft of approaches will be needed, ranging from public education to selective taxation, if other less restrictive measures fail. Restriction of inappropriate advertising, especially to children, and clear food labelling are also important approaches.

Q: Are current food labelling practices adequate?

A: Required back-of-pack labelling, which typically includes information about total sugars and sometimes added sugars, can be difficult for consumers to understand. Such labels are often difficult to read and provide information on the amount of free sugars contained in 100 g of the product or in a typical serving, which is unhelpful when the package contains more than one serving. Clear front-of-pack labelling such as the traffic light labelling may be more helpful for the consumer. It enables consumers to quickly determine whether a product has been assessed as being high, medium or low in free sugars and other nutrients, for example. Such a labelling system should be consistent and compulsory in each country and – better still – internationally. We are still far away from such an approach and, of course, many foods are not packaged and have no labels.

Q: In some countries there have been arguments over conflicts of interest: government advisers on nutrition committees having close ties to industry. Are conflicts of interest unavoidable?

A: In some countries it may be difficult to recruit expert advisers who have no connection to the food industry. When expert advisers do have such connections, it is essential that they declare any potential conflicts of interest that could affect their ability to provide impartial advice. These potential conflicts of interest may range from the fairly trivial, such as having provided informal and unpaid advice, to the serious, such as personal financial gain or major financial benefit to the institution where the expert is employed. The responsible authority must decide whether the conflict is serious enough to exclude that individual from providing independent advice. It is vital that the entire process is transparent. WHO has very strict rules to ensure that individuals with any significant conflict of interest are excluded from providing advice on the development of guidelines and recommendations.

Q: What would be the pitfalls for governments keen to follow the draft WHO recommendations on free sugars?

A: There are none, unless they receive funding from the food industry and risk losing that support. But the long-term potential health gains overall should by far outweigh such short-term considerations. Some public health experts argue that governments could use the revenues derived from taxes on sugar-sweetened beverages for health purposes. Most countries now accept the health risks associated with tobacco and many are taking tough measures to reduce smoking. Recently WHO Director-General Margaret Chan said that Big Food was the next Big Tobacco. It’s vital that national governments heed the Director-General’s warning, and develop policies which create an environment that encourages healthy food choices.

